# Alkaline autofermentation of *Nostoc muscorum* enhances organic acid production and antimicrobial activity against *Salmonella enterica* and *Campylobacter jejuni*

**DOI:** 10.3389/fmicb.2026.1830932

**Published:** 2026-04-22

**Authors:** Mohana Krishnan Neelakrishnan, Brahmaiah Pendyala, Akhil Rautela, Ankit Patras

**Affiliations:** Department of Food and Animal Sciences, Tennessee State University, Nashville, TN, United States

**Keywords:** antimicrobial activity, autofermentation, *Campylobacter jejuni*, cyanobacteria, *Nostoc muscorum*, organic acids, *Salmonella enterica*

## Abstract

Foodborne pathogens such as *Salmonella enterica* and *Campylobacter jejuni* remain leading causes of bacterial gastroenteritis and are frequently associated with poultry production systems. Increasing restrictions on antibiotic use in animal agriculture have intensified the need for sustainable antimicrobial alternatives. Cyanobacteria represent a promising resource for producing antimicrobial metabolites; however, their antimicrobial potential has primarily been explored using solvent extracts derived from fresh biomass. While alkaline autofermentation and organic acid production in cyanobacteria have been previously reported, including in our earlier work, the antimicrobial potential of autofermentation-derived aqueous extracts, which are primarily composed of organic acids, remains largely unexplored. This study establishes, for the first time, a dual-product autofermentation framework in *Nostoc muscorum* that enhances antimicrobial activity through the generation of organic acid–rich extracts and metabolite-enriched residual biomass. Autofermentation was conducted at pH 8.4 and 10.3, and organic acid production and antimicrobial activity against *S. enterica* and *C. jejuni* were evaluated using minimum inhibitory concentration assays. Alkaline autofermentation significantly enhanced metabolite production, yielding 12.31 g L^−1^ total organic acids after 24 h at pH 10.3, a 1.6-fold increase compared with pH 8.4. Butyric acid was the dominant fermentation product, reaching 9.11 g L^−1^ under alkaline conditions (pH 10.3), and contributed substantially to antimicrobial activity. Organic acid–rich extracts inhibited both pathogens, while methanolic extracts from autofermented biomass exhibited greater potency than fresh biomass extracts. These findings demonstrate that alkaline autofermentation enhances the antimicrobial potential of cyanobacterial biomass and highlight *N. muscorum* as a promising platform for developing antibiotic-free pathogen control strategies in poultry production systems.

## Graphical abstract

**Figure:**
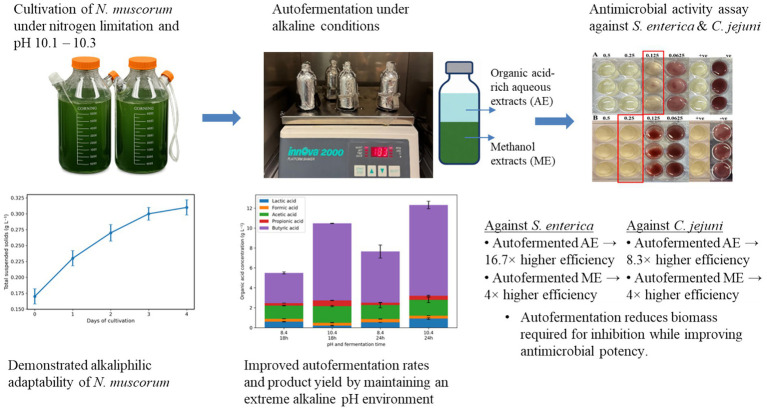

## 1 Introduction

Foodborne bacterial pathogens remain a major public health concern worldwide. Among them, *Salmonella enterica* and *Campylobacter jejuni* are recognized as leading causes of bacterial gastroenteritis, responsible for millions of infections annually (Zhang et al., 2006; Van Immerseel, 2007). These pathogens are commonly associated with poultry-derived foods and are capable of colonizing the gastrointestinal tract of birds without causing overt disease, leading to persistent environmental contamination and transmission through the food chain (O'Bryan et al., 2022; Bhunia, 2008). In the United States alone, *Salmonella* spp. infections account for approximately 1.35 million illnesses annually, resulting in an estimated 26,500 hospitalizations and 420 deaths (CDC, 2022). Similarly, *Campylobacter* spp. infections cause approximately 1.3 million illnesses each year, with poultry products identified as a major source of human exposure (Fischer et al., 2024). The continued prevalence of these pathogens highlights the need for effective antimicrobial strategies that can reduce pathogen burden and improve food safety.

Historically, antibiotics have been widely used in animal production systems not only to treat bacterial infections but also as growth promoters to improve feed efficiency and animal performance. However, the emergence and global spread of antimicrobial resistance have raised significant concerns regarding the long-term sustainability of antibiotic-dependent strategies (Zhang et al., 2006; Funk and Gebreyes, 2004). Consequently, regulatory restrictions and increased awareness of antimicrobial resistance have accelerated the search for alternative approaches that can maintain animal health and productivity while reducing antibiotic usage. Natural antimicrobial compounds, including organic acids, phytogenic compounds, probiotics, and bioactive peptides, have emerged as promising alternatives that can simultaneously suppress pathogenic bacteria and support intestinal health (Khan et al., 2022).

Among these alternatives, organic acids such as butyrate, acetate, propionate, and lactate play a particularly important role. These short-chain fatty acids exhibit antimicrobial activity by lowering environmental pH, disrupting bacterial membrane integrity in their undissociated form, and interfering with intracellular metabolic processes (Khan et al., 2022; Casewell et al., 2003; Ayalew et al., 2022). In addition to their direct antibacterial effects, organic acids have been widely reported to improve gut health by modulating intestinal microbiota, enhancing nutrient digestibility, strengthening intestinal barrier function, and promoting epithelial cell development. These combined effects contribute to improved growth performance and disease resistance in animal production systems.

Cyanobacteria (blue-green algae), rapidly growing photosynthetic microorganisms, represent an increasingly recognized microbial source of bioactive metabolites with potential antimicrobial and health-promoting applications (Encarnação et al., 2015; Altermann et al., 2006; Pathak et al., 2022). Cyanobacteria produce a diverse array of secondary metabolites with reported antibacterial, antifungal, antiviral, and immunomodulatory activities (Pathak et al., 2022; Rippka et al., 1979; Bhadury and Wright, 2004; Dahms et al., 2006; Jaki et al., 2000; Kajiyama et al., 1998; Gustafson et al., 1989; Gerwick et al., 1994; Alvarez-Sánchez et al., 2024). Several compounds isolated from cyanobacterial genera such as *Nostoc*, *Fischerella*, *Lyngbya*, and *Scytonema* have demonstrated inhibitory activity against pathogenic bacteria, including *Salmonella* spp. and *Campylobacter* spp. (Cock and Cheesman, 2023; Carpine and Sieber, 2021). In addition to secondary metabolites, cyanobacterial cells accumulate substantial amounts of intracellular glycogen, which can serve as a carbon reserve for fermentative metabolism.

Under dark, anoxic conditions, cyanobacteria can undergo autofermentation, a metabolic process in which intracellular glycogen is converted into short-chain organic acids such as butyrate, acetate, and lactate (Demirkaya et al., 2023; Hasunuma et al., 2016). Unlike conventional microbial fermentation processes that rely on external substrates or microbial inoculation, autofermentation utilizes endogenous carbon reserves within the cells, providing a self-sustaining metabolic pathway for organic acid production. Previous studies have shown that strongly alkaline conditions (pH > 10) can accelerate this metabolic conversion by increasing glycogen degradation and enhancing organic acid formation (Pendyala et al., 2020). These autofermentation-derived metabolites possess well-established antimicrobial properties and may also contribute to improved intestinal health through mechanisms similar to dietary acidifiers.

Importantly, while cyanobacterial metabolites have been widely investigated for antimicrobial properties, most studies have focused on solvent extracts derived from fresh biomass rather than autofermentation-derived products. In this study, we introduce a dual-product autofermentation framework in *Nostoc muscorum*, in which autofermentation generates (i) an organic acid–rich aqueous extract and (ii) a metabolite-enriched residual biomass, thereby enhancing antimicrobial potency and improving overall biomass utilization. This work represents the first systematic investigation of alkaline autofermentation in *N. muscorum* under extreme alkaline conditions (pH 10.3) and establishes a direct link between autofermentation-derived metabolites and antimicrobial activity. Previous studies have demonstrated that short-chain organic acids such as butyrate, acetate, and propionate are effective against foodborne pathogens including *Salmonella* spp. and *Campylobacter* spp., primarily through intracellular acidification and disruption of metabolic processes (Ricke, 2003). In particular, butyrate and acetate have been widely reported to reduce *Salmonella enterica* colonization and inhibit *Campylobacter* spp. survival under acidic conditions (Van Immerseel et al., 2006; Beier et al., 2019). These findings highlight the relevance of organic acid–based antimicrobial strategies for controlling enteric pathogens in food and animal production systems. Therefore, the objective of this study was to evaluate the antimicrobial activity of autofermented *N. muscorum* against *S. enterica* and *C. jejuni*, while also assessing the effect of alkaline autofermentation on organic acid production and biomass-to-organic acid conversion efficiency. By integrating metabolic conversion with enhanced antimicrobial functionality, this work advances the development of cyanobacterial autofermentation as a microbial bioprocess for enhancing the antimicrobial potential of cyanobacterial biomass, with applications in pathogen control and future animal health and nutrition strategies.

## 2 Materials and methods

### 2.1 Cultivation of cyanobacteria

The freshwater non-toxic cyanobacterial strain *N. muscorum* (UTEX 2209) was obtained from the UTEX Culture Collection of Algae (Austin, TX, USA). Cultures were grown photoautotrophically in batch mode using 8 L glass photobioreactors (Corning) with a working volume of 6 L. The cyanobacteria were cultivated in nitrate-limited BG-11 medium containing 0.824 mM nitrate, which was intentionally reduced to promote the accumulation of intracellular glycogen. The medium was supplemented with 20 mM sodium bicarbonate, and the initial pH was adjusted to 10.1 using sodium carbonate to enhance inorganic carbon availability and maintain alkaline conditions favorable for biomass production, as previously reported (Vadlamani et al., 2018). These conditions were also selected to evaluate the alkaliphilic adaptability of *N. muscorum* and to generate biomass suitable for subsequent autofermentation experiments under strongly alkaline conditions. Cultivation was performed under a 12 h light/12 h dark photoperiod. Temperature was maintained at 25 ± 1 °C throughout the cultivation period. Cultures were continuously mixed with aeration to ensure uniform light distribution and efficient gas exchange. Culture purity was periodically verified by microscopic examination to confirm the absence of contaminating microorganisms. Illumination was provided by warm blue and red LED lamps at a surface intensity of 200 μmol m^−2^ s^−1^ measured using model LI-250A light meter, Li-Cor Biosciences, Lincoln, NE.

The average light intensity within the photobioreactor was estimated by integrating the incident light spectrum with the wavelength-dependent absorbance of the cyanobacterial culture. The emission spectrum of the LED light source (400–700 nm) was measured using the Ocean Optics Spectrometer (QE Pro series, Ocean Optics, Dunedin, FL, USA), which was equipped with an irradiance probe. Spectral irradiance (μW cm^−2^ nm^−1^) was then converted to spectral photon flux density (μmol m^−2^ s^−1^ nm^−1^) using wavelength-dependent energy-to-photon conversion.

The absorbance spectrum of the culture, measured at 1 cm path length, was scaled to the effective optical path length of the reactor (11.7 cm). Wavelength-specific light attenuation was incorporated using the Beer–Lambert law, and the depth-averaged spectral photon flux was calculated as:

$${\bar{I}}_{\lambda }=I_{\lambda ,0}\cdot \frac{1-10^{-A_{\lambda }L}}{A_{\lambda }L\ln(10)}$$

where $I_{\lambda ,0}$ is the incident spectral photon flux, $A_{\lambda }$ is the absorbance per cm, and L is the optical path length (cm).

The average internal photosynthetically active radiation (PAR) was obtained by integrating the attenuated spectral photon flux across the 400–700 nm range. Based on this approach, the incident surface PAR was calculated to be approximately 200 μmol m^−2^ s^−1^, while the average internal PAR within the reactor was estimated to be 72.9 μmol m^−2^ s^−1^.

### 2.2 Harvesting of cyanobacterial biomass

Cyanobacterial biomass was harvested by centrifugation to separate the cells from the culture medium. Cultures were transferred into sterile centrifuge bottles and centrifuged at 3,839 × g for 10 min at room temperature (22–25 °C) using a Thermo Scientific Sorvall Legend XTR centrifuge. These conditions enabled efficient biomass recovery while minimizing cell damage. Following centrifugation, the supernatant was carefully decanted and the compact cyanobacterial pellet was retained. The harvested biomass was subsequently used for washing, autofermentation experiments, and metabolite extraction (Pendyala et al., 2020).

### 2.3 Alkaline autofermentation conditions

The harvested cyanobacterial biomass pellet was resuspended in carbonate buffer systems prepared to establish two initial pH conditions for autofermentation. For pH 8.4, the biomass was suspended in 40 mM sodium bicarbonate (NaHCO₃), which provided buffering capacity under mildly alkaline conditions. For pH 10.3, a carbonate buffer (125 mM) consisting of 30:70 NaHCO₃/Na₂CO₃ (molar ratio) was used to establish and maintain strongly alkaline conditions. These two pH levels were selected to compare autofermentation performance under mild (pH 8.4) and extreme alkaline conditions (pH 10.3), based on previous studies demonstrating the influence of alkaline environments on cyanobacterial metabolism and carbon utilization (Vadlamani et al., 2018). It was hypothesized that strongly alkaline conditions would enhance autofermentation rates, increase organic acid yield, and potentially alter product distribution. The carbonate buffering system served both to stabilize the pH during fermentation and to supply inorganic carbon. Autofermentation experiments were conducted in sterile 160 mL serum vials containing 50 mL of biomass suspension (Pendyala et al., 2020). To establish anoxic conditions, the vials were sparged with argon gas to remove dissolved oxygen from both the liquid phase and the headspace. Immediately after sparging, the vials were sealed with butyl rubber septa and aluminum crimp caps to prevent oxygen entry. To suppress photosynthetic activity, the sealed vials were wrapped in aluminum foil and incubated in darkness at 30 ± 0.5 °C with continuous agitation at 100 rpm. Samples were collected at predetermined time intervals and centrifuged to separate the biomass from the liquid phase. The filtered supernatants were subsequently stored for organic acid analysis.

### 2.4 Preparation of aqueous and methanolic extracts

After autofermentation, cultures were centrifuged at 3,839 × g for 10 min at room temperature (22–25 °C) to separate liquid and solid fractions. The clarified supernatant was collected as the aqueous extract, which contained organic acids and other polar metabolites. For methanolic extraction, both fresh (non-autofermented) and autofermented biomass were used. Fresh biomass was obtained after cultivation and harvesting as described in Section 2.2, without undergoing autofermentation. Both fresh and autofermented biomass samples were dried at 40 °C until constant weight, as described in Section 2.5.1, and then ground into a fine powder to ensure homogeneity. For extraction, 1 g of dried biomass was suspended in 20 mL of a methanol–chloroform mixture (1,1, v/v). The suspension was sonicated for 1 h and incubated overnight at 4 °C to facilitate the extraction of intracellular metabolites. The mixture was then centrifuged at 3,839 × g for 10 min at room temperature (22–25 °C), and the supernatant containing cyanobacterial metabolites was filtered through a 0.45 μm syringe filter to remove particulate material. The filtrate was concentrated under reduced pressure using a rotary evaporator and dried at 40 °C to remove residual solvent. The dried extract was stored at −20 °C until use. Prior to antimicrobial testing, the extract was reconstituted in sterile methanol at defined concentrations.

### 2.5 Analytical methods

#### 2.5.1 Determination of total suspended solids

Total suspended solids (TSS) were determined following the National Renewable Energy Laboratory protocol (NREL Method) (Van Wychen and Laurens, 2013). Briefly, 50 mL of culture was filtered through a pre-weighed 0.2 μm membrane filter. The filter with retained biomass was dried at 40 °C to minimize thermal degradation of biomass constituents until constant weight was achieved, verified by repeated weighing until no further mass change was observed. The final biomass concentration was calculated gravimetrically and expressed as grams of dry biomass per liter (g L^−1^).

#### 2.5.2 Quantification of organic acids

Liquid samples (2 mL) were withdrawn at defined intervals and immediately centrifuged at 3839 × g rpm for 10 min. The clarified supernatants were filtered through 0.45 μm syringe filters and stored at −20 °C prior to analysis. Organic acids were analyzed using an HPLC system (Shimadzu, Japan) equipped with an autosampler and photodiode array detector. Separation was achieved using a Bio-Rad fermentation monitoring column (150 × 7.8 mm, 9 μm) with an H^+^ guard column (30 × 4.6 mm). The mobile phase consisted of 0.005 N H₂SO₄ delivered at a flow rate of 1 mL min^−1^. The injection volume was 10 μL and detection was performed at 210 nm. Quantification was based on external calibration curves prepared using standard organic acids (0.625–5 μg mL^−1^), including lactic acid (Fisher Chemical, CAS: 50-21-5), formic acid (Thermo Scientific, CAS: 64-18-6), acetic acid (Fisher Chemical, CAS: 64-19-7), propionic acid (TCI, CAS: 79-09-4), and butyric acid (Thermo Scientific, CAS: 107-92-6).

#### 2.5.3 Antimicrobial activity assay

The antimicrobial activity of cyanobacterial extracts was evaluated against two bacterial pathogens: *S. enterica* (ATCC 10398) and *C. jejuni* (ATCC BAA-1062), obtained from the American Type Culture Collection (ATCC, Manassas, VA, USA). *S. enterica* was cultured in Tryptic Soy Broth (TSB) at 37 °C under aerobic conditions, whereas *C. jejuni* was cultured in TSB at 37 °C under microaerophilic conditions. Bacterial cultures were grown to approximately 10^8^–10^9^ CFU mL^−1^ prior to antimicrobial testing. After autofermentation, the pH of the aqueous extract was adjusted to 4.9 using 1 M HCl to simulate the gut pH of the animals. The relationship between pH and the dissociated/undissociated forms of organic acids was estimated using the Henderson–Hasselbalch equation (pH = pKa + log[A^−^]/[HA]). Minimum inhibitory concentration (MIC) assays were performed using a broth microdilution method following Clinical and Laboratory Standards Institute guidelines (Eloff, 1998). Bacterial suspensions were adjusted to approximately 5 × 10^5^ CFU mL^−1^ and 100 μL of the suspension was added to each microtiter well containing serial dilutions of the extracts. Sterility and growth controls were included for each strain. Plates were incubated at 37 °C for 24 h. After incubation, 40 μL of a 0.4 mg mL^−1^ solution of 2-(4-iodophenyl) -3-(4-dinitrophenyl) -5-phenyltetrazolium chloride (INT) was added as a microbial growth indicator, and plates were incubated for an additional 30 min. The MIC was defined as the lowest extract concentration showing no visible growth. Ampicillin (Sigma-Aldrich) served as the positive control, while solvent-only wells served as negative controls.

### 2.6 Statistical analysis

All experiments were conducted in biological triplicate unless otherwise stated. Organic acid concentrations and antimicrobial activity data were expressed as mean ± standard deviation. Statistical analyses were performed using SAS statistical computing environment (SAS Institute Inc., Cary, NC, USA). Organic acid production differences between pH 8.4 and 10.3 autofermentation conditions were evaluated using one-way analysis of variance (ANOVA) followed by Tukey’s multiple comparison test. Differences were considered statistically significant at *p* < 0.05.

## 3 Results

### 3.1 Growth and biomass accumulation of *N. muscorum*

**Figure 1:**
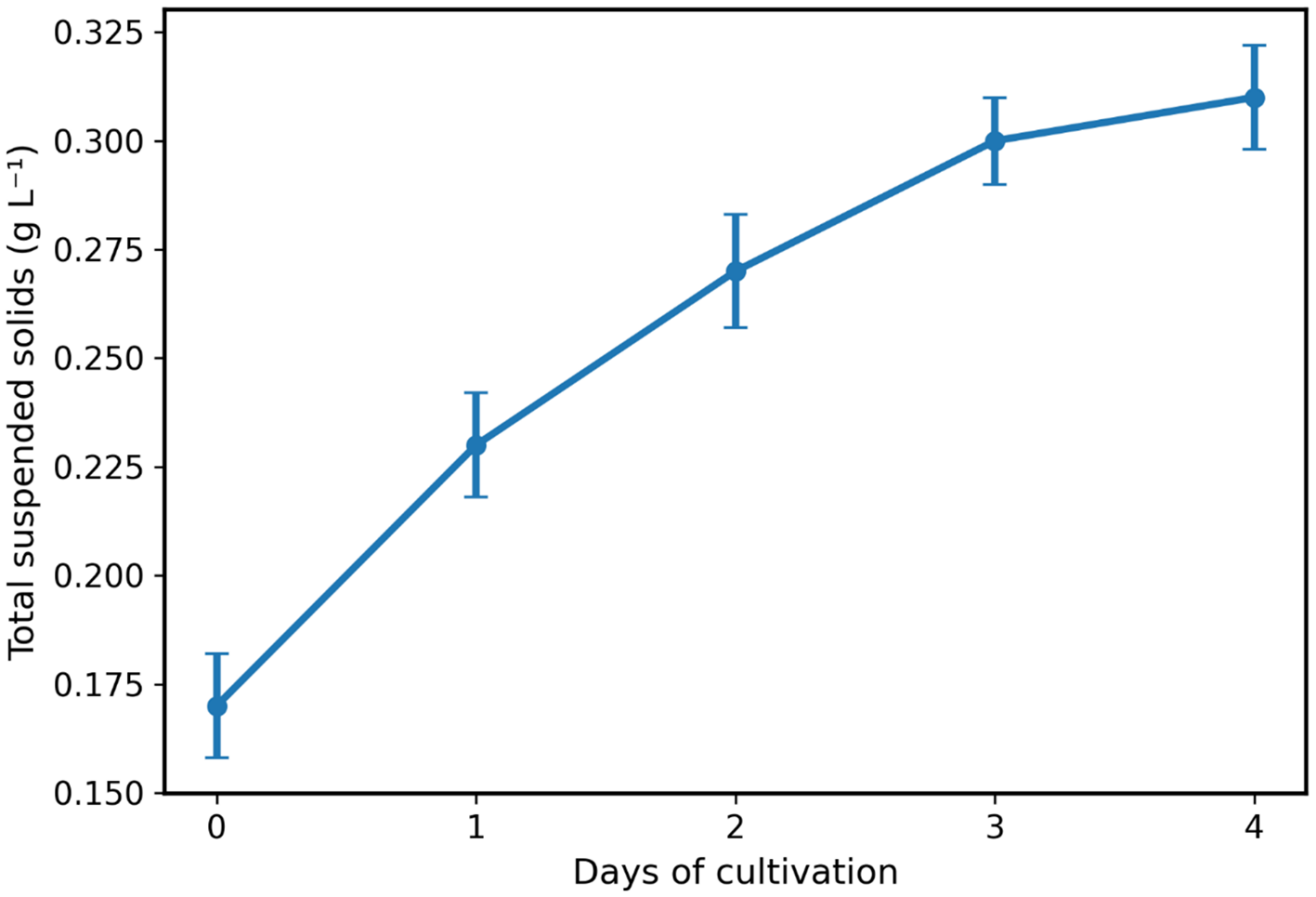
Biomass accumulation of *N. muscorum* during alkaline cultivation.

The cyanobacterial strain *N. muscorum* was cultivated under alkaline conditions in nitrate-limited BG-11 medium using controlled photobioreactor conditions. Biomass accumulation was monitored using total suspended solids (TSS) as an indicator of biomass concentration (Figure 1). At the beginning of the monitored period (day 0), the culture exhibited a TSS concentration of 0.17 g L^−1^. Biomass increased progressively during the following days, reaching 0.23 g L^−1^ by day 1 and 0.27 g L^−1^ by day 2, indicating active biomass production under the applied cultivation conditions. Continued growth resulted in TSS values of 0.30 g L^−1^ on day 3 and 0.31 g L^−1^ by days 4 and 5. Although biomass accumulation continued throughout the monitored period, the rate of increase gradually declined during the later stages of cultivation. The biomass harvested after cultivation was subsequently used for autofermentation experiments.

### 3.2 Effect of alkaline conditions on autofermentation and organic acid production

**Figure 2:**
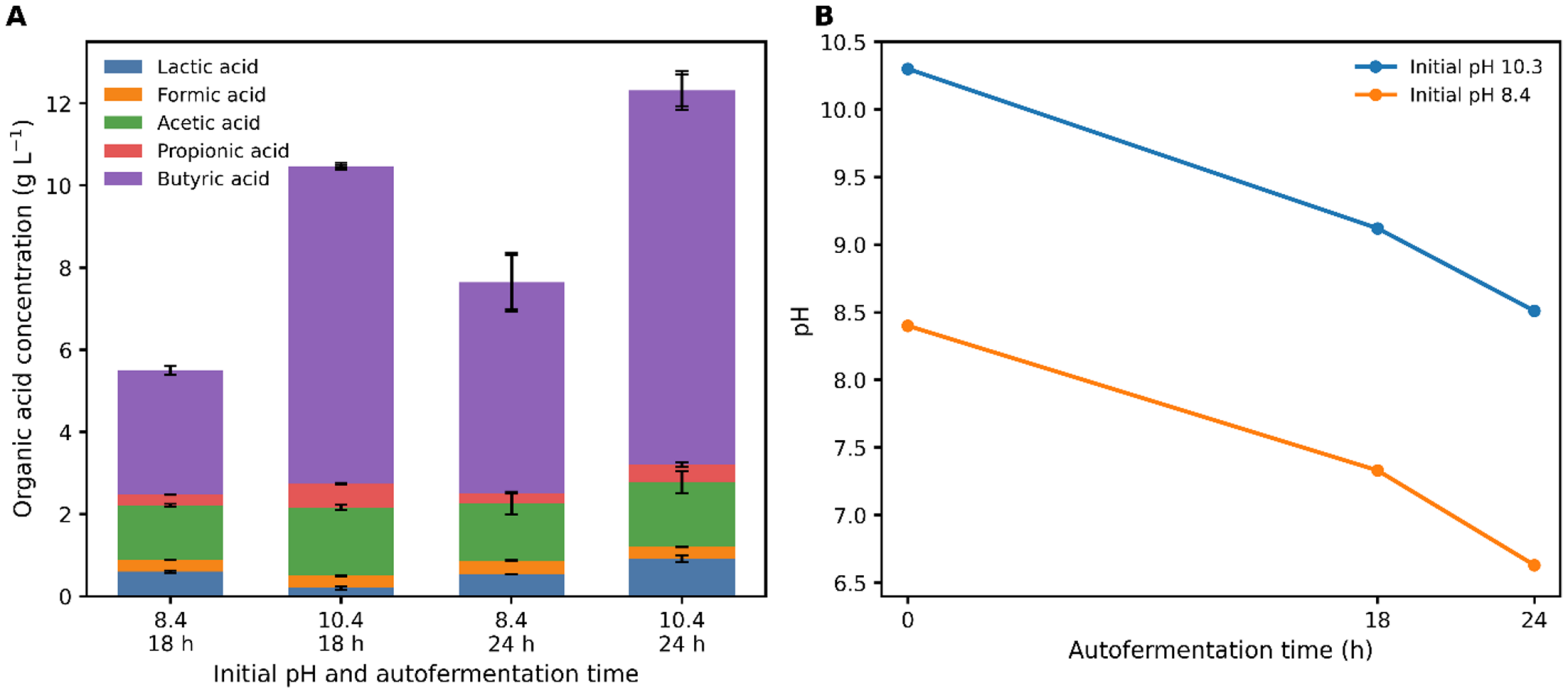
Organic acid production during alkaline autofermentation of *N. muscorum*. **(A)** Stacked bars represent concentrations of individual organic acids produced after 18 h and 24 h of autofermentation at pH 8.4 and pH 10.4. **(B)** Changes in pH during autofermentation under the two initial pH conditions (8.4 and 10.4) over the 24 h incubation period.

To evaluate the influence of alkaline conditions on fermentative metabolism, harvested *N. muscorum* biomass was subjected to autofermentation under dark, anoxic conditions at mild and extreme alkaline pH levels (pH 8.4 and pH 10.3) using a carbonate buffer system. Fermentation was initiated at an initial biomass concentration of 20 g L^−1^, and organic acid production was monitored over time. Organic acid production increased during fermentation and was strongly influenced by the initial pH (Figure 2A). At 18 h, fermentation conducted at pH 10.3 produced approximately 10.47 g L^−1^ of total organic acids, whereas fermentation at pH 8.4 generated approximately 5.5 g L^−1^, representing nearly a 1.9-fold increase under the higher pH condition. This difference remained evident at 24 h, when total organic acid production reached 12.31 g L^−1^ at pH 10.3, compared with 7.65 g L^−1^ at pH 8.4 (Table 1), corresponding to an approximately 1.6-fold increase in total acid yield. The difference in total organic acid production between the two pH conditions was statistically significant (*p* < 0.05). During autofermentation, the pH of the cultures decreased gradually due to the accumulation of organic acids (Figure 2B). Under the initial pH 10.3 condition, the pH declined to 9.12 after 18 h and further to 8.51 after 24 h of fermentation. Similarly, cultures initiated at pH 8.4 exhibited a reduction in pH to 7.33 and 6.63 after 18 h and 24 h, respectively. Overall, these results indicate that strongly alkaline conditions enhance the *N. muscorum* autofermentation rates and yields of organic acid fermentation products.

**Table 1:** Organic acid production by *N. muscorum* during autofermentation (24 h) at different alkaline conditions.

Organic acid	pH 8.4 (g/L)	pH 10.3 (g/L)
Lactic acid	0.54 ± 0.01^b^	0.91 ± 0.07^a^
Formic acid	0.33 ± 0.01^a^	0.28 ± 0.01^a^
Acetic acid	1.40 ± 0.28^a^	1.59 ± 0.27^a^
Propionic acid	0.24 ± 0.01^b^	0.42 ± 0.06^a^
Butyric acid	5.14 ± 0.66^b^	9.11 ± 0.38^a^
Total organic acids	7.65 ± 0.72^b^	12.31 ± 0.48^a^

Values represent mean ± standard deviation (*n* = 3). Different superscript letters (a, b) within a row indicate significant differences between treatments (*p* < 0.05).

Analysis of the organic acid profile showed that fermentation produced several short-chain organic acids, including lactic, formic, acetic, propionic, and butyric acids (Table 1). Among these, butyric acid was the predominant fermentation product under both conditions and increased significantly at pH 10.3 (9.11 ± 0.38 g L^−1^) compared with pH 8.4 (5.14 ± 0.66 g L^−1^) (p < 0.05). Similarly, lactic acid and propionic acid concentrations were significantly higher under extreme alkaline conditions (*p* < 0.05). In contrast, formic acid and acetic acid showed no significant differences between the two pH conditions (*p* > 0.05). The results demonstrate that extreme alkaline conditions not only enhance total organic acid production but also influence product distribution, with a marked increase in butyrate as the dominant metabolite.

### 3.3 Antimicrobial activity of autofermented *N. muscorum* biomass extracts

The antimicrobial activity of the extracts obtained from autofermented *Nostoc muscorum* biomass was evaluated against two major foodborne pathogens, *S. enterica* and *C. jejuni*, using a broth microdilution minimum inhibitory concentration (MIC) assay. Both aqueous extracts, containing fermentation-derived organic acids, and methanolic extracts, containing intracellular metabolites, were tested.

#### 3.3.1 Antimicrobial activity of aqueous extract

The aqueous extract obtained after autofermentation contained a mixture of short-chain organic acids dominated by butyric acid (Table 1). Prior to antimicrobial testing, the extract pH was adjusted to 4.9 to simulate the gut pH of the animals, which ensured that the organic acids remained predominantly in their undissociated form, which facilitates membrane diffusion and enhances antimicrobial efficacy. Table 2 show speciation of organic acids produced by *N. muscorum* at pH 4.9 used for MIC evaluation. Serial dilutions of the extract were prepared to determine the minimum concentration required to inhibit bacterial growth (Table 2). The MIC endpoints were visually confirmed using the INT colorimetric assay. Wells containing actively growing bacteria developed a pink coloration, whereas wells with inhibited bacterial growth remained colorless, clearly indicating the inhibitory concentrations of the extract. The aqueous extract inhibited *S. enterica* at a 0.125 × concentration, corresponding to approximately 1.54 g L^−1^ total organic acids. At lower concentrations (0.0625 × concentration, 0.77 g L^−1^ total organic acids), bacterial growth was observed, indicating that this concentration was insufficient for inhibition. In contrast, *C. jejuni* required a higher concentration of organic acids for growth inhibition. Complete inhibition was observed at a 0.25 × concentration, corresponding to approximately 3.08 g L^−1^ total organic acids, while bacterial growth persisted at lower concentrations. These results indicate that *C. jejuni* exhibited greater tolerance to the organic acid mixture than *S. enterica* under the tested conditions.

**Table 2:** Speciation of organic acids produced by *N. muscorum* at pH 4.9 during MIC evaluation.

Organic acid	pKa	Acid form (HA) (g/L)	Organic acid salt	Base form (A^−^) (g/L)
Lactic acid	3.86	0.08	Lactate	0.84
Formic acid	3.75	0.02	Formate	0.26
Acetic acid	4.76	0.67	Acetate	0.92
Propionic acid	4.87	0.2	Propionate	0.22
Butyric acid	4.82	4.14	Butyrate	4.98
Total		5.1		7.21

#### 3.3.2 Antimicrobial activity of methanolic extracts

Methanolic extracts were prepared to evaluate antimicrobial compounds present within the cyanobacterial biomass. The antimicrobial activity of extracts derived from fresh biomass and autofermented biomass was compared (Table 3). The methanolic extract obtained from fresh *N. muscorum* biomass required a concentration of 4 mg mL^−1^ to inhibit both *S. enterica* and *C. jejuni*. In contrast, the extract derived from autofermented biomass exhibited substantially greater antimicrobial potency. The autofermented extract inhibited *S. enterica* at concentrations below 1 mg mL^−1^, whereas *C. jejuni* was inhibited at approximately 2 mg mL^−1^.

**Table 3:** Minimum inhibitory concentration (MIC) of aqueous and methanolic extracts derived from fresh and autofermented *N. muscorum* biomass against *S. enterica* and *C. jejuni*.

Extract Type	Concentration (mg/mL)	*S. enterica*	*C. jejuni*
Autofermented aqueous extract (total organic acids)			
	6.16	−	−
	3.08	−	−
	1.54	−	+
	0.77	+	+
Fresh methanol extract (extract powder)			
	1	+	+
	2	+	+
	3	+	+
	4	−	−
	5	−	−
	6	−	−
Autofermented methanol extract (extract powder)			
	1	−	+
	2	−	−
	3	−	−
	4	−	−
	5	−	−
	6	−	−

Concentrations for aqueous extracts represent total organic acids (mg/mL) obtained by dilution (2, 4, 8, 16 times) of the autofermented extract. Concentrations for methanolic extracts represent methanol extract powder (mg/mL). ‘–’ indicates no bacterial growth (inhibition), ‘+’ indicates visible bacterial growth. Bold rows indicate MIC values.

Autofermentation substantially improved the antimicrobial efficiency of *N. muscorum* by generating two active product streams from the same starting biomass: an organic acid–rich aqueous fraction and an enriched residual biomass fraction (Table 4). When antimicrobial activity was normalized to the amount of starting cyanobacterial biomass required for inhibition, both autofermented fractions outperformed the fresh methanolic extract. The aqueous fraction showed the greatest improvement, requiring only 4.8 mg biomass/mL against *S. enterica* and 9.6 mg biomass/mL against *C. jejuni*, compared with 80 mg biomass/mL for the fresh methanolic extract. The methanolic extract from autofermented biomass also showed improved potency, requiring only 20 mg biomass/mL for inhibition of both pathogens.

**Table 4:** Comparison of antimicrobial efficiency of fresh and autofermented *N. muscorum* extracts.

Extract type	*S. enterica*		*C. jejuni*	
	MIC (mg/mL)	Overall biomass (mg/mL)	MIC (mg/mL)	Overall biomass (mg/mL)
Fresh methanol extract	4.0	80	4.0	80
Autofermented aqueous extract (organic acids)	2.4	4.8	4.8	9.6
Autofermented methanol extract	1.0	20	2.0	20

Biomass required was calculated based on the amount of cyanobacterial biomass needed to produce the inhibitory extract concentration.

## 4 Discussion

This study demonstrates that alkaline autofermentation of *N. muscorum* enhances organic acid production and generates extracts with antimicrobial activity against the foodborne pathogens *S. enterica* and *C. jejuni*. These results highlight the potential of cyanobacterial metabolic processes as a source of bioactive metabolites for pathogen control and the development of antibiotic-free antimicrobial strategies.

During cultivation, nitrogen limitation likely played a key role in promoting intracellular glycogen accumulation. Under nitrogen-deficient conditions, cyanobacteria reduce protein and pigment synthesis while redirecting carbon flux toward storage compounds such as glycogen (Forchhammer, 2004; Flores and Herrero, 2005). This metabolic adaptation is particularly important for autofermentation, as glycogen serves as the primary substrate for organic acid production under dark, anoxic conditions. In addition, the gradual reduction in biomass productivity observed at later stages of cultivation may be attributed to light limitation within the photobioreactor. As biomass density increased, self-shading likely reduced light penetration, thereby limiting photosynthetic activity and slowing further growth. Such effects are commonly reported in dense cyanobacterial cultures and can influence biomass productivity in photobioreactor systems. Furthermore, the ability of *N. muscorum* to maintain stable growth under strongly alkaline conditions (pH 10.3) highlights its alkaliphilic adaptability. Cultivation under elevated pH is advantageous for large-scale bioprocessing, as it can reduce contamination risks and promote selective growth of alkaliphilic microorganisms (Pendyala et al., 2020; Vadlamani et al., 2018; Pendyala et al., 2021).

A major finding of this work was the strong influence of alkaline conditions on autofermentative metabolism. The selection of alkaline autofermentation was motivated by limitations in previous studies (Gaffron and Rubin, 1942; Klein and Betz, 1978; Kreuzberg, 1984; Gfeller and Gibbs, 1984; Meuser et al., 2009; Catalanotti et al., 2013), where autofermentation of photosynthetic microorganisms was primarily conducted under circumneutral pH conditions (pH 6–7.5) and resulted in mixed product profiles, including organic acids, alcohols, and gaseous metabolites. These systems often exhibited low autofermentation rates and limited control over product distribution. In contrast, pH is a critical parameter that can regulate metabolic pathways by influencing intracellular pH, proton gradients, membrane potential, and ATP-dependent processes. Based on this understanding, we hypothesized that alkaline conditions could redirect metabolic flux toward organic acid production. Our previous work confirmed that extreme alkaline conditions (pH > 10) significantly enhance fermentation rates and improve organic acid yields by stimulating glycogen catabolism and increasing cellular energy demand associated with pH homeostasis (Pendyala et al., 2020). Under alkaline conditions, autofermentation appears to favor organic acid–dominated metabolism while suppressing competing pathways such as alcohol and gas production, thereby improving carbon conversion efficiency. Unlike conventional acidogenic fermentation systems that rely on heterotrophic microorganisms and external substrates, alkaline autofermentation in *N. muscorum* utilizes intracellular glycogen reserves under conditions aligned with its alkaliphilic physiology. The high organic acid yields observed in this study further support the effectiveness of alkaline autofermentation as a strategy to enhance metabolite production and overcome limitations of neutral pH fermentation systems.

The enhanced acid production observed under alkaline conditions suggests that elevated pH stimulates glycogen catabolism and redirects carbon flux toward autofermentation products. Decreased rate of autofermentation from 18 to 24 h at initial pH 10.3 may be attributed to limitation of stored glycogen. Similar metabolic responses have been reported in other cyanobacteria exposed to environmental stress conditions, where metabolic pathways shift toward the production of reduced metabolites (Stal and Moezelaar, 1997; Hagemann, 2011). Among the metabolites detected in this study, butyric acid represented the dominant fermentation product. This observation is significant because butyrate is widely recognized for its antimicrobial properties and beneficial effects on intestinal health. Short-chain fatty acids such as butyrate have been shown to inhibit pathogenic bacteria while supporting beneficial gut microbiota and maintaining intestinal epithelial integrity (Salvi and Cowles, 2021; Peng and Biswas, 2017). The predominance of butyrate, therefore, represents a key functional feature of the autofermentation system observed in this study.

The antimicrobial assays confirmed that the organic acid–rich extracts derived from autofermented biomass effectively inhibited both *S. enterica* and *C. jejuni*. Organic acids can diffuse across bacterial membranes and subsequently dissociate inside the cytoplasm, leading to intracellular acidification, disruption of membrane potential, and inhibition of essential metabolic processes (Rebelo et al., 2023; Hirshfield et al., 2003). These mechanisms collectively contribute to the observed antibacterial effects. The two pathogens exhibited different sensitivities to the organic acid mixture, with *C. jejuni* showing greater tolerance to acidic conditions. This observation is consistent with previous studies indicating that *Campylobacter* species possess stress response systems that enable survival during transient acid exposure (Beier et al., 2019; Kim et al., 2021). Nevertheless, both pathogens were effectively inhibited by the autofermentation-derived extracts, indicating the broad antimicrobial potential of the organic acid mixture produced by *N. muscorum*. Autofermentation also improved the antimicrobial efficiency of the cyanobacterial biomass. Previous studies have reported antimicrobial activity of *Nostoc* spp. primarily using methanolic crude extracts, with reported MIC values typically in the range of approximately 0.125–1.5 mg mL^−1^ depending on the organism and extract composition (Li and Guo, 2017; Kaushik et al., 2008). However, these studies report antimicrobial efficacy based solely on extract concentration, and do not provide information on biomass-to-extract conversion or biomass-equivalent antimicrobial efficiency. In contrast, the present study demonstrates that autofermentation significantly enhances antimicrobial efficiency by reducing the biomass required for pathogen inhibition. This enhanced activity likely results from the breakdown of intracellular glycogen and its conversion into organic acids during autofermentation, which contributes to the concentration of antimicrobial metabolites. In addition, partial disruption or weakening of the cyanobacterial cell wall during autofermentation may increase the accessibility of intracellular bioactive compounds, further enhancing antimicrobial efficacy. These results further indicate that autofermentation enhances antimicrobial activity through dual mechanisms: (i) conversion of intracellular glycogen into highly active organic acids and (ii) improved antimicrobial efficiency of the residual biomass.

From an applied perspective, these findings highlight the potential of cyanobacterial autofermentation as a sustainable approach for producing natural antimicrobial metabolites. Short-chain fatty acids such as butyrate, acetate, and propionate are widely used as feed additives due to their ability to improve gut health and suppress enteric pathogens. The autofermentation process developed in this study enables the direct generation of these compounds from cyanobacterial biomass without the need for external microbial fermentation systems. Importantly, for practical applications, the use of whole autofermented biomass comprising both the organic acid–rich aqueous fraction and the residual biomass offers a solvent-free and integrated approach. The aqueous fraction can serve as a natural preservative and primary antimicrobial component, while the residual biomass provides additional bioactive metabolites and nutrients that may contribute to enhanced antimicrobial activity. This integrated system improves overall biomass utilization and eliminates concerns associated with solvent residues. Furthermore, this approach aligns with the development of sustainable and scalable feed additive strategies. Future studies will focus on evaluating metabolite bioavailability during digestion and exploring food-grade extraction or direct biomass application methods to facilitate practical implementation in animal production systems.

## 5 Conclusion

This study demonstrates that alkaline autofermentation of *N. muscorum* provides an effective metabolic approach for converting cyanobacterial biomass into organic acid–rich extracts with antimicrobial activity. Autofermentation under dark, anoxic conditions at elevated pH (10.3) resulted in enhanced metabolite production, yielding 12.31 g L^−1^ total organic acids, representing a 1.6-fold increase compared with pH 8.4. Among the metabolites, butyric acid was the dominant product (9.11 g L^−1^), contributing substantially to antimicrobial efficacy. The organic acid–rich aqueous extracts effectively inhibited *S. enterica* and *C. jejuni*, while methanolic extracts from autofermented biomass exhibited improved antimicrobial potency compared with extracts from non-autofermented biomass. Autofermentation also reduced the biomass required for pathogen inhibition, indicating enhanced functional efficiency of cyanobacterial metabolites. Importantly, this study establishes a dual-product bioprocess framework in which autofermentation generates (i) an organic acid–rich aqueous fraction with strong antimicrobial activity and (ii) a metabolite-enriched residual biomass with additional antibacterial potential. This integrated approach improves overall biomass utilization and supports the development of solvent-free, sustainable antimicrobial systems. Overall, these findings highlight the potential of alkaline autofermentation as a scalable and sustainable platform for producing bioactive metabolites from cyanobacterial biomass, with promising applications in pathogen control and future animal health strategies. Future studies should focus on *in vivo* validation, process scale-up, and evaluation of metabolite bioavailability under practical application conditions.

### 5.1 Limitations and future perspectives

Although the results demonstrate the antimicrobial potential of fermentation-derived extracts from *N. muscorum*, this study was conducted under controlled laboratory conditions using *in vitro* assays. The antimicrobial activity observed in vitro may not fully reflect the complexity of microbial interactions and environmental conditions within animal production systems. Future research should therefore evaluate the efficacy of these fermentation-derived metabolites in *in vivo* animal studies to determine their impact on pathogen colonization, gut microbiota, and overall animal health. In addition, further work is needed to optimize fermentation conditions and scale-up the process to assess its feasibility for larger-scale biotechnological applications. Comprehensive metabolomic characterization of fermentation products may also help identify additional bioactive compounds contributing to antimicrobial activity. Such studies will improve understanding of cyanobacterial fermentation metabolism and support the development of sustainable microbial platforms for producing natural antimicrobial compounds.

## Data Availability

The original contributions presented in the study are included in the article/supplementary material, further inquiries can be directed to the corresponding author.
